# The hypoxic tumor microenvironment in vivo selects the cancer stem cell fate of breast cancer cells

**DOI:** 10.1186/s13058-018-0944-8

**Published:** 2018-03-06

**Authors:** Hoon Kim, Qun Lin, Peter M. Glazer, Zhong Yun

**Affiliations:** Department of Therapeutic Radiology, Yale University School of Medicine, P. O. Box 208040, New Haven, CT 06520-8040 USA

**Keywords:** AKT, Breast cancer cell, Cancer stem cell, Cell fate, Hypoxia, PI3K, Tumor microenvironment, Xenograft

## Abstract

**Background:**

Tumor hypoxia is an independent prognostic factor associated with poor patient survival. Emerging evidence suggests that hypoxia can potentially maintain or enhance the stem cell phenotype of both normal stem cells and cancer cells. However, it remains to be determined whether cell fate is regulated in vivo by the hypoxic tumor microenvironment (TME).

**Methods:**

We established a hypoxia-sensing xenograft model to identify hypoxic tumor cell in vivo primarily using human breast cancer cell lines MDA-MB-231 and MCF7. Hypoxic tumor cells were identified in situ by fluorescence of green fluorescence protein. They were further isolated from xenografts, purified and sorted by flow cytometry for detailed analysis of their stem cell characteristics.

**Results:**

We have found that hypoxic tumor cells freshly isolated from xenografts contain increased subpopulations of tumor cells with cancer stem cell (CSC)-like characteristics. The CSC characteristics of the hypoxic tumor cells are further enhanced upon re-implantation in vivo, whereas secondary xenografts derived from the non-hypoxic tumor cells remain similar to the primary xenografts. Interestingly, the phenotypes exhibited by the hypoxic tumor cells are stable and remain distinctively different from those of the non-hypoxic tumor cells isolated from the same tumor mass even when they are maintained under the same ambient culture conditions. Mechanistically, the PI3K/AKT pathway is strongly potentiated in the hypoxic tumor cells and is required to maintain the CSC-like phenotype. Importantly, the differential cell fates between hypoxic and non-hypoxic tumor cells are only found in tumor cells isolated from the hypoxic TME in vivo and are not seen in tumor cells treated by hypoxia in vitro alone.

**Conclusions:**

These previously unknown observations suggest that the hypoxic TME may promote malignant progression and therapy resistance by coordinating induction, selection and/or preferential maintenance of the CSC-like phenotype in tumor cells.

**Electronic supplementary material:**

The online version of this article (10.1186/s13058-018-0944-8) contains supplementary material, which is available to authorized users.

## Background

Tumor hypoxia, a hallmark of tumor microenvironment (TME), is an independent prognostic factor for advanced disease progression and poor patient survival [1–3]. As an emerging paradigm, hypoxia has the potential to regulate cell fate and differentiation, especially the cellular properties associated with stem cells [4–8]. These observations provide important insights into the role of TME in the regulation of malignant tumor progression from the perspective of cancer stem cells (CSCs). CSCs are defined as a distinct subpopulation of tumor cells with unlimited self-renewal and tumor-initiating potential [9, 10]. Current studies have shown that CSCs are likely to be the major cause of therapy resistance and tumor recurrence [9, 11]. Similar to normal stem cells, maintenance and differentiation of CSCs are also subject to regulation by both genetic and environmental or niche factors [10, 12–14]. Studies have shown that the hypoxic TME is strongly associated with increased cancer cell “stemness”. In clinical tumor specimens, tumor cells located in hypoxic regions appear poorly differentiated and express stem-cell-associated genes [15, 16]. Poorly differentiated pancreatic cancer cells show strong nuclear accumulation of hypoxia-inducible factor 1α (HIF-1α) protein [17]. Higher HIF-α protein levels are also found in the stemcell-like tumor cells in neuroblastomas [18, 19] or gliomas [20]. When maintained under in vitro hypoxic conditions, tumor cells exhibit enhanced clonogenicity [21–23] and can activate an embryonic stem cell-like transcription program in a HIF-dependent manner [24]. Recent studies using three-dimensional culture systems have reported hypoxia-associated cell-type plasticity [25] and CSC heterogeneity [26]. These data suggest that tumor hypoxia is likely to have a direct and strong impact on the fate of cancer cells, especially CSCs. However, it remains to be clearly determined whether the CSC fate is differentially regulated by the hypoxic TME in vivo.

In this study, we have developed a hypoxia-sensing xenograft model using human breast cancer cell lines and have made several new discoveries with regard to cell fate determination by the hypoxic TME. First, we have obtained direct evidence that the CSC-like subpopulation is significantly enriched in the enhanced green fluorescent protein (EGFP)^+^ hypoxic population. Second, the CSC characteristics continue to be enhanced upon re-implantation of the EGFP^+^ hypoxic cells, suggesting the CSC-like properties displayed by the hypoxic tumor cells are stable and continue to be enriched. Consistently, the phenotypes exhibited by the EGFP^+^ hypoxic tumor cells remain stably distinct from those of the EGFP^−^ non-hypoxic tumor cells isolated from the same tumor mass even when they are maintained under the same ambient culture condition. Third, the PI3K/AKT pathway is strongly potentiated in the EGFP^+^ hypoxic tumor cells and is required to maintain the CSC-like phenotype. Interestingly, the cell fate differences between hypoxic and non-hypoxic tumor cells are found only in the tumor cells isolated from the hypoxic TME in vivo, but not in tumor cells treated by hypoxia in vitro alone. These previously unrecognized observations suggest that tumor hypoxia may promote malignant progression and therapy resistance by differential selection for and/or maintenance of the CSC-like tumor cells in the hypoxic TME.

## Methods

### Generation of the hypoxia-sensing cell lines

MDA-MB-231 and MCF7 human breast cancer cells (American Type Culture Collection (ATCC)) were transfected with 5HRE/GFP plasmid (a gift from Martin Brown and Thomas Foster, Addgene plasmid # 46926) [27] and selected with 500 μg/ml of G418. For positive selection, cells were incubated at 1% O_2_ in a hypoxia chamber (Invivo 400) for 24 h and EGFP^+^ cells were collected by flow cytometry. For negative selection, the sorted EGFP^+^ cells were then incubated under normal tissue culture conditions and EGFP^−^ cells were sorted by flow cytometry. Three rounds of positive and negative selections were used to establish the hypoxia-sensing cell lines. All cell lines are authenticated using the short tandem repeat (STR) method.

### Xenografts

MDA-MB-231/HRE-EGFP and MCF7/HRE-EGFP cells were mixed with growth factor-depleted Matrigel (1:1) and then injected either orthotopically into the mammary fat pads or ectopically under the skin in the upper back of female athymic mice (6–7 weeks of age) at a concentration of 1–2 × 10^6^ cells per injection site. For tumor takeandgrowth curve analysis, 10 × 10^3^ cells were injected per site. When the tumor size reached approximately 800 mm^3^, tumors were excised for isolation of tumor cells.

### Tumor cell dissociation and purification

Excised xenograft tumors were minced and incubated at 37 °C for 2 h in a dissociation medium containing 10% fetal calf serum, 0.5 U/ml dispase (07913, STEMCELL Technologies), 5 mg/ml Collagenase Type IV (CLS-4, Worthington Biochemical), and 100 U/ml penicillin streptomycin. Red blood cells were removed by incubation with a red blood cell lysis buffer containing 0.8% NH_4_Cl for 10 min on ice. Host mouse cells were depleted using a Mouse Cell Depletion Kit (130–104-694, Miltenyi Biotec.)

### Analysis of cell surface marker by flow cytometry

All antibodies used for flow cytometry were purchased from eBiosciences (ThermoScientific). For CD24 and CD44 double stain, anti-CD24-PE-Cyanine 7 (1:20, #25–0247-42) and anti-CD44-eFlour 450 (1:50, #48–0441-82) were used with mouse IgGκ-PE-Cyanine 7 (1:20, #25–4714-42) and rat IgG2b-eFlour 450 as IgG controls (1:50, #48–4031). For CD44 and CD49f double stain, anti-CD44-eFlour 450 (1:50) and anti-CD49f-PE-Cyanine 7 (1:20, #25–0495-82) were used with rat IgG2b-eFlour 450 and mouse IgGκ-PE-Cyanine 7 as IgG controls. For human epithelial cell adhesion molecule (EPCAM) stain, anti-CD326-PE-Cyanine 7 (1:20, #25–9326-41) was used with mouse IgGκ-PE-Cyanine 7 as IgG control. The BD LSR II flow cytometer was used for fluorescence-activated cell sorting (FACS). The instruments were calibrated daily. FACS data were analyzed using the FlowJo™ software.

### Flow-assisted cell sorting

Human tumor cells were isolated from xenografts and separated from the host mouse cells. After filtration through a 40-μm cell strainer, tumor cells were then sorted into the hypoxic (EGFP^+^, approximately the top 30%) and non-hypoxic (EGFP^−^, approximately the bottom 30%) fractions using the BD FACSAria™ II.

### Side-population analysis

Cells were suspended at 1 × 10^6^ cells/ml in pre-warmed Dulbecco’s modified Eagle’s medium (DMEM, Life Technologies) with 2% fetal calf serum (FCS) and 10 mM HEPES buffer. Hoechst 33342 (ThermoScientific) was added at a final concentration of 5 μg/ml in the presence or absence of verapamil hydrochloride (50 μM, Sigma-Aldrich). After incubation at 37 °C for 90 min, cells were washed with ice-cold FACS buffer containing 3% FCS and 10 mM HEPES, resuspended in the FACS buffer, and filtered through a 40-μm cell strainer. Propidium iodide was added at a final concentration of 2 μg/ml before FACS to gate viable cells. FACS analyses were done using the LSRII (BD Bioscience). The Hoechst 33342 dye was excited at 350 nm and its fluorescence was dual-wavelength analyzed (blue, 440 nm; red, 650–670 nm).

### Detection of hypoxic regions in xenografts

Hypoxyprobe™-1 (pimonidazole HCl, Hypoxyprobe, Inc) was injected intraperitoneally 2 h before xenograft tumor isolation at a dose of 60 mg/kg bodyweight. Tumors were excised, fixed in formalin, and cryopreserved in optimum cutting temperature compound (OCT). Tumor sections (7 μm thick) were incubated with anti-pimonidazole rabbit IgG1 antibody (PAB2627AP, Hypoxyprobe, Inc.) and then a fluorescently labeled anti-rabbit IgG antibody. Nuclei were counter stained with Hoechst 33342. The fluorescence stains were examined under a fluorescence microscope (Zeiss ZX10 Imager M2).

### Clonogenic assay

The clonogenic assay is based on our previously published protocols [22, 28]. Briefly, tumor cells were plated at 600 cells/well for MDA-MB-231 cells and at 1000 cells/well for MCF7 cells in 6-well plates and incubated for 10 to 14 days. Colonies were stained with crystal violet. Plating efficiency (PE) = numbers of colonies (≥50 cells/colony) divided by numbers of cells plated × 100%.

### Tumor sphere formation assay

The tumor sphere formation assay has been described in our previous publication [22]. Briefly, tumor cells were incubated as a single-cell suspension in a tumor sphere medium containing DMEM-F12, 3:1 (Invitrogen), 2% B27 supplement, 40 ng/ml basic fibroblast growth factor (bFGF), and 20 ng/ml epidermal growth factor (EGF), and plated into tissue culture dishes pre-coated with polyhydroxyethylmethacrylate (polyHEMA, Sigma-Aldrich). After incubation for 4 to 6 days, the numbers of tumor spheres were counted under a microscope.

### Tumor cell invasion assay

Invasion of tumor cells was determined using trans-well chambers with polycarbonate membranes of 8-μm pore size according to the manufacturer’s instructions. The dividing membranes were pre-coated with 50 μl 15% Matrigel solution in serum-free medium. Freshly sorted tumor cells isolated from xenograft tumors were suspended in a culture medium containing 0.5% FBS and loaded (10 × 10^3^ cells per well) into the upper chamber of the trans-well plates. The lower chambers were filled with a culture medium containing 10% fetal bovine serum (FBS). After incubation for 48 h, cells migrating to the underside of the membrane were stained with Diff-Quik™ Stain Set (B4132-1A, Siemens) and cells remaining on the upper surface of the membrane were removed with a cotton swab. The number of migrated cells on each membrane was counted in 20 random fields at ×200 magnification under a light microscope.

### Wound healing assay

Tumor cells were allowed to grow to 100% confluence in 60-mm dishes. Wound tracks were created by manually scraping the cell monolayer with a P1000 pipet tip. After incubation for 12 and 24 h, the gap filling was examined under a light microscope. Wound healing is quantitatively shown as percent gap fill.

### Western blot analysis

Antibodies to the following antigens were used for western blots: phospho-AKT-S473 (#4060, 1:5000, Cell Signaling), total AKT (#4691, 1:5000, Cell Signaling), phospho-ERK1/2-T202/T204 (#4377, 1:10,000, Cell Signaling), total ERK1/2 (#9102, 1:10,000, Cell Signaling), phospho-PI3Kp85 (#4228, 1:1000, Cell Signaling), phospho-mTOR-S2448 (#5536, 1:1000, Cell Signaling), HIF-1α (#14179, 1:1000, Cell Signaling), HIF-2α (#NB100–122, 1:1000, Novus Biologicals), β-actin (#A5316, 1:10,000, Sigma Aldrich), and vinculin (ab18058, 1:10,000, Abcam). Protein bands were visualized using ECL substrates (ThermoFisher Scientific, #34080) and imaged using the Kodak X-OMAT 2000A.

### Real-time quantitative reverse transcription PCR (RT-qPCR)

Total cellular RNA was isolated with the TRizol reagent (Invitrogen). First-strand cDNA was synthesized using the High Capacity cDNA Reverse Transcription Kit (Thermo Fisher Scientific). RT-qPCR was performed on StepOne Plus (Applied Biosystems) using iTaq Universal SYBR Green Supermix (Bio-Rad) under the following conditions: initiation at 95 °C × 30 s, 40 cycles at 95 °C × 15 s, and 60 °C × 60 s. The housekeeping gene HPRT was used as a control for normalization. Specificity of the primers was confirmed by a single peak on the dissociation curve. For CD44, TGCCGCTTTGCAGGTGTAT (forward) and GGCCTCCGTCCGAGAGA (reverse); for CD24, AAACAACAACTGGAACTTCAAGTAACTC (forward) and GGTGGTGGCATTAGTTGGATTT (reverse); for GLUT1, GATTGGCTCCTTCTCTGTGG (forward) and TCAAAGGACTTGCCCAGTTT (reverse); for LOX1, GAACACAGGAACATCATCCTG (forward) and ACGCAGCACAGTCCTTGGTT (reverse); for HPRT, TATGGCGACCCGCAGCCCT (forward) and CATCTCGAGCAAGACGTTCAG (reverse).

### Global gene expression analysis

Total cellular RNA was isolated with RNeasy® Mini Kit (74,104, Qiagen) and treated with DNase I for 10 min. Global gene expression profile analysis was done using Affymetrix HTA microarrays at the Yale Center for Genomic Analysis. The hybridization patterns and signal intensities were analyzed and interpreted using the Affymetrix GeneChip Expression Console.

### Statistics

Microarray data were analyzed using analysis of variance (ANOVA) by Yale Center for Analytical Sciences (YCAS). Two-group comparisons were analyzed using the two-tailed Student’s *t* test (GraphPad Prizm 7). A significant difference was declared if the *p* value was < 0.05.

## Results

### The cancer stem cell-like population of tumor cells is enriched in the hypoxic TME in vivo

The cell fate regulation of tumor cells by the TME is critical for understanding clonal heterogeneity and malignant progression but remains to be fully investigated. We have established two hypoxia-sensing tumor models by stably expressing a hypoxia-responsive transcription enhancer element (five tandem repeats of HRE or 5XHRE)-driven destabilized d2EGFP construct [27] in human breast cancer cell line MDA-MB-231 and MCF7, respectively. These two cell lines represent the two most common types of breast cancer, i.e. the basal type (MDA-MB-231) and the luminal type (MCF7). The HRE-EGFP reporter gene is transcriptionally activated by hypoxia-inducible transcription factor (HIF)-1 and/or HIF-2 [29]. Similar strategies have been used in other tumor models [30, 31] to identify hypoxic cells. We generated the hypoxia-reporter cell lines using three rounds of negative and positive selection to ensure low background under normoxic conditions and robust induction of EGFP under hypoxic conditions (Additional file 1). We also confirmed that HIF accumulation occurring during hypoxia treatment is readily reversible in the ambient air in the sorted *ex vivo* tumor cells from xenografts generated by our hypoxia-sensing cancer cell lines (Additional file 2, panel A).

**Fig. 1:**
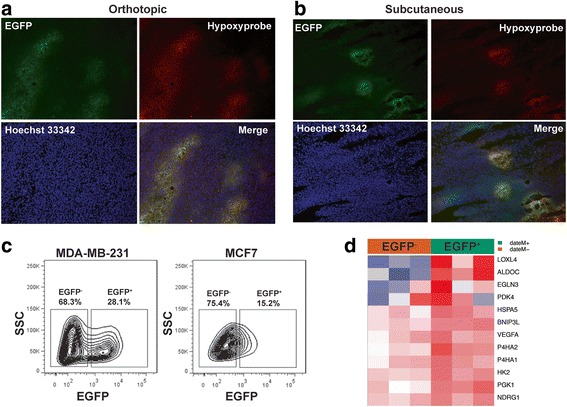
The hypoxia-sensing human breast cancer xenograft model. **a**, **b** MDA-MB-231 cells stably expressing the HRE-EGPF reporter gene are implanted either orthotopically in mammary fat pads (**a**) or ectopically in the hind back (**b**) of female athymic mice. Hypoxic regions are visualized by immunostaining of the Hypoxyprobe (red). Expression of the hypoxia reporter gene is shown by fluorescence of enhanced green fluorescent protein (EGFP). Nuclei are counterstained with Hoechst 33342. **c** The hypoxic populations from the MDA-MB-231/HRE-EGFP and MCF7/HRE-EGFP xenografts, respectively, are analyzed by fluorescence-activated cell sorting. **d** Microarray analysis shows that expression of a panel of commonly observed hypoxia-induced genes is significantly upregulated in the EGFP^+^ cells freshly isolated from the MDA-MB-231/HRE-EGFP xenografts, compared to the EGFP^−^ cells from the same xenografts (*n* = 3; analysis of variance, *p* < 0.05). SSC, side scatter

Next, we generated both orthotopic (mammary fat pads) and ectopic (subcutaneous sites) xenografts in female athymic *nu/nu* mice to examine the distribution of the EGFP^+^ tumor cells in the tumor microenvironment. The hypoxic regions in xenografts were independently identified using the bioreductive compound pimonidazole HCl (Hypoxyprobe-1). As shown in Fig. 1a, the EGFP^+^ MDA-MB-231 cells were primarily localized in the pimonidazole-positive regions in both orthotopic and subcutaneous xenografts. Similar results were found in MCF7-derived xenografts (Additional file 1).

We next purified the human tumor cells from xenografts (Additional file 3) and separated the EGFP^+^ and EGFP^−^ cells by flow cytometry (Fig. 1c). Consistent with the literature [32], the basal-like MDA-MB-231 cell-derived xenografts contained a larger hypoxic population than the xenografts derived from the luminal-like MCF7 cells (Fig. 1c). Using global gene expression analysis, we found that a panel of commonly observed hypoxia-inducible genes [33–35], including *BNIP3L*, *EGLN3*, *LOXL4* and *P4HA1*, were significantly upregulated in the EGFP^+^ MDA-MB-231 tumor cells, compared to the EGFP^−^ cells (Fig. 1d). Collectively, these data demonstrate that these HRE-EGFP-modified breast cancer cell lines can serve as a reliable hypoxia-sensing model to identify hypoxic tumor cells in xenografts.

**Fig. 2:**
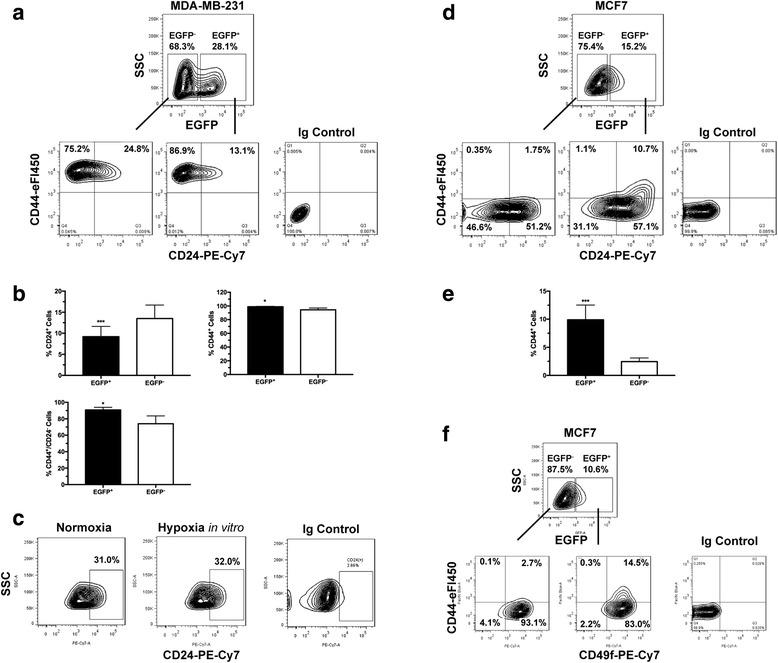
Cancer stem cell (CSC)-like cells are enriched in the hypoxic populations freshly isolated from xenografts. Tumor cells are enzymatically dissociated and isolated from either the MDA-MB-231/HRE-EGFP (**a**-**c**) or MCF7/HRE-EGFP (**d**-**f**) xenografts. Stem cell characteristics are evaluated by fluorescence-activated cell sorting (FACS) for the expression of CSC-associated surface markers CD24, CD44 and CD49f. Representative FACS plots are shown in **a**, **c**, **d** and **f**. Quantitative population analyses are shown in **b** (*n* = 4–5; *p < 0.05, ***p < 0.001, Student’s *t* test) and **e** (n = 4; ***p < 0.001, Student’s *t* test). These results are confirmed by three or more independent experiments. EGFP, enhanced green fluorescent protein; SSC, side scatter

The cell fate or the state of differentiation of tumor cells is thought to be under the influence of the tumor microenvironment [36–38]. In particular, the impact of hypoxia on the regulation of CSC-associated phenotypes and functions has been reported [7, 39–41]. However, definitive in vivo evidence has remained elusive. We hypothesized that the hypoxic tumor microenvironment can alter tumor cell fate with preference for cancer stem-cell-like phenotypes. Using FACS-based single-cell analysis, we examined the stem cell fate of freshly isolated tumor cells using common cancer stem cell markers [42], including CD44, CD24, and CD49f for breast CSCs. Tumor cells isolated from the basal-like MDA-MB-231 cell-derived xenografts are predominantly CD44^+^ (Fig. 2a, b). The *ex vivo* EGFP^+^ or hypoxic population contains an average of 8% CD24^+^ cells, whereas the non-hypoxic or EGFP^−^ population contains much higher numbers of CD24^+^ cells at approximately 13% (Fig. 2a, b), suggesting that the hypoxic TME favors tumor cells with the CD24^−/low^ CSC-like characteristics. Similar results are obtained from both orthotopic and subcutaneous xenografts. The differential expression of the CD44 and CD24 surface markers was independently confirmed by qRT-PCR using the freshly sorted cells (Additional file 3). These results are consistent with the findings that the CSCs of basal-like breast cancer cells tend to display the CD44^+^/CD24^−^ phenotype [43, 44]. Consistent with these observations, there was a slight increase in the side population (SP) with low retention of Hoechst 33342 in the freshly sorted hypoxic tumor cells (Additional file 3) although MDA-MB-231 cells generally contain a very small SP.

Among other CSC markers, we found the expression of the CSC marker CD133 and aldehyde dehydrogenase (ALDH) activity were not significantly different in MDA-MB-231 cells isolated from different tumor microenvironments (data not shown), suggesting the hypoxic TME exerts specific effects on the CD44/CD24 pathways. Interestingly, hypoxia in vitro does not significantly affect the CD24^+^ cell population (Fig. 2c). These findings underscore the unique ability of hypoxic TME in vivo to enhance and/or maintain the CSC phenotype, which cannot be recapitulated simply by exposure to hypoxia in vitro.

In contrast to the basal-like MDA-MB-231 cell-derived tumors, the hypoxic TME had a different impact on the cell fate of the luminal-like MCF7-derived xenografts. The non-hypoxic (EGFP^−^) MCF7 cells exoko vivo had very low levels of CD44 (Fig. 2d, e). However, there was a significant increase in the numbers of the CD44^+^ cells in the hypoxic (EGFP^+^) MCF7 tumor cell population with the predominant increase of the CD44^+^/CD24^+^ population (Fig. 2d, e). Interestingly, it has been shown that the CD24^+^ MCF7 cells exhibit higher proliferation and stronger invasion than their CD24^−^ counterparts [45]. Clinically, CD24 overexpression is significantly associated with unfavorable outcomes in patients with luminal A breast cancers [46] or with breast tumors of intermediate-grade differentiation [47]. Furthermore, the CD44^+^/CD49f^+^ population is also strongly increased among the hypoxic (EGFP^+^) MCF7 tumor cells (Fig. 2f). Our data suggest that the CSC-like population of the luminal-type MCF7 cells are likely to be characterized by the CD44^+^/CD24^+^ phenotype, which is strongly enhanced in the hypoxic TME. Collectively, these data provide direct ex vivo evidence demonstrating enrichment of the CSC-like breast tumor cells in the hypoxic TME.

### The *ex vivo* hypoxic tumor cells possess aggressive CSC-like properties

**Fig. 3:**
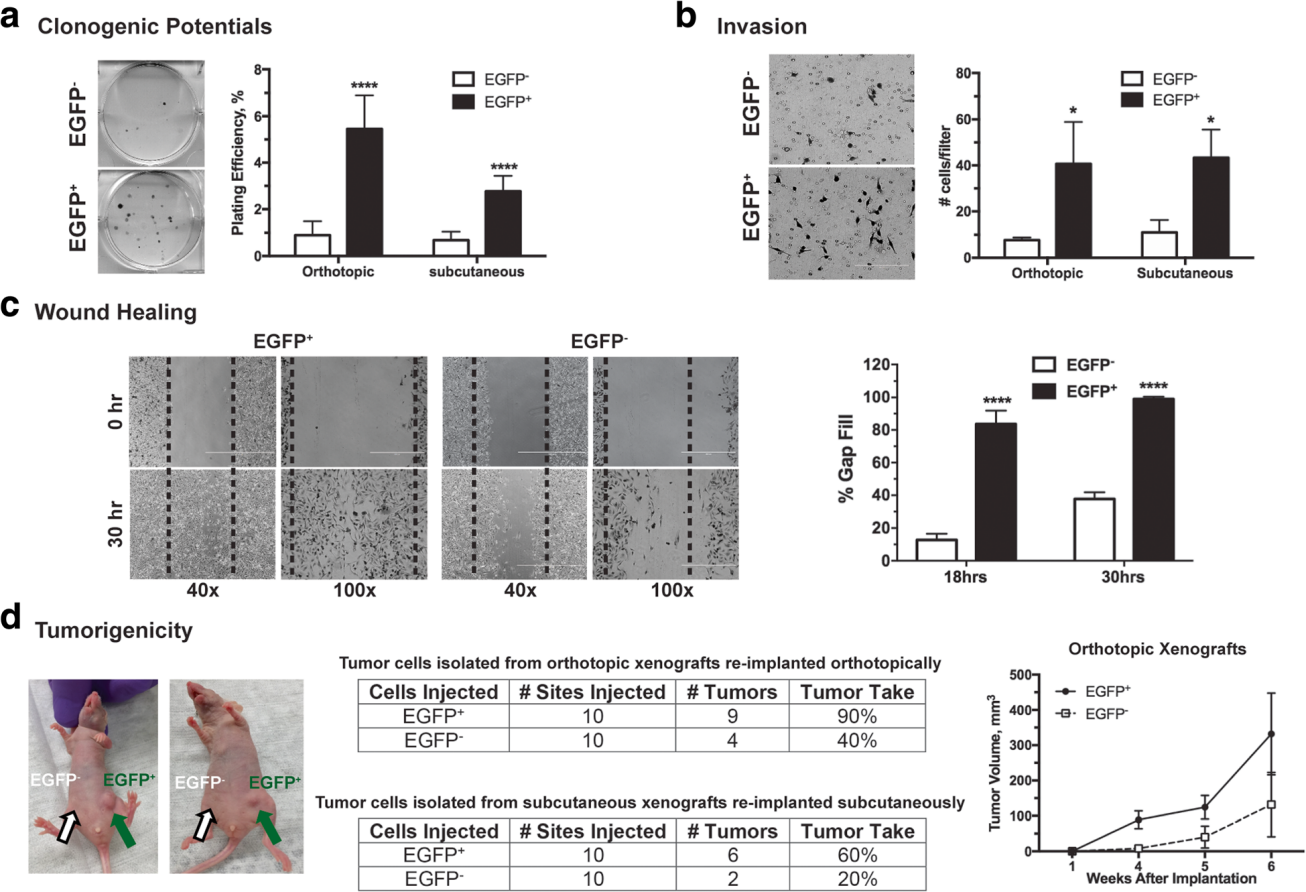
The ex vivo hypoxic tumor cells possess properties functionally associated with self-renewal and tumorigenic potentials. Tumor cells are enzymatically dissociated and isolated from the MDA-MB-231/HRE-EGFP xenografts. After sorting into the enhanced green fluorescent protein (EGFP)^+^ and EGFP^−^ populations, tumor cells were plated for *in vitro* assays (**a**-**c**) or directly re-implanted in athymic mice (**d**). Detailed experimental conditions are described in “Methods”. **a** Clonogenic potential (*n* = 6, ***p < 0.001, Student’s *t* test). **b** Tumor cell invasion (n = 3, *p < 0.05, Student’s *t* test). **c** Wound healing potentials (*n* = 5, *p < 0.001, Student’s *t* test). **d** Tumorigenic potentials *in vivo* are primarily reflected by percent tumor take (the ability of implanted cells to produce a tumor)

Using robust in vitro functional assays, we examined the TME-dependent stemness of the EGFP^+^ hypoxic and EGFP^−^ non-hypoxic tumor cells. Although the tumor sphere formation assay has been widely used to assess the self-renewal potential of CSC-like cells in some tumors or tumor cell lines, MDA-MB-231 tumor cells, however, do not form tumor spheres. We instead used the in vitro clonogenic assay to examine the ability of an individual tumor cells to establish a colony. We have found, using the freshly isolated 4T1/HRE-EGFP mouse mammary tumor cells (Additional file 4), that the clonogenic potential is closely correlated with the ability to grow as tumor spheres. As shown in Fig. 3a, the EGFP^+^ hypoxic tumor cells produced significantly more colonies than the EGFP^−^ tumor cells did when plated at clonal densities (<1 cell/mm^2^). The increased self-renewal ability and clonogenic potential of the ex vivo hypoxic MDA-MB-231 tumor cells were observed in both orthotopic and subcutaneous xenografts, suggesting a strong association with the hypoxic TME.

The ex vivo hypoxic MDA-MB-231 tumor cells also demonstrated strong invasion and migration. In contrast to non-hypoxic tumor cells, we found that the freshly sorted EGFP^+^ hypoxic MDA-MB-231 tumor cells invaded more readily through the Matrigel barrier (Fig. 3b). Furthermore, the ex vivo EGFP^+^ hypoxic MDA-MB-231 tumor cells were capable of healing a wounded monolayer in cell culture much more rapidly and efficiently than their non-hypoxic counterparts (Fig. 3c). These data demonstrate that the ex vivo EGFP^+^ hypoxic tumor cells possess enhanced self-renewal and clonogenic potential and highly invasive characteristics.

We further examined the tumorigenic potential of the ex vivo hypoxic and non-hypoxic MDA-MB-231 tumor cells, respectively, by implanting the freshly sorted EGFP^+^ and EGFP^−^ tumor cells in athymic mice. As shown in Fig. 3d, the *ex vivo* EGFP^+^ orthotopic tumor cells formed new xenografts at a tumor-take rate of 90% versus 40% for the EGFP^−^ tumor cells. Similarly, the tumor-take rate was 60% for the freshly isolated EGFP^+^ subcutaneous tumor cells versus 20% for the EGFP^−^ tumor cells. The secondary xenografts originating from the ex vivo EGFP^+^ orthotopic tumor cells also grew at a higher rate than did xenografts formed from the EGFP^−^ tumor cells (Fig. 3d). These results demonstrate that the hypoxic TME can select tumor cells with biological properties associated with aggressive and CSC-like phenotypes, including enhanced self-renewal, pronounced invasiveness and robust tumor-initiating potential.

### The CSC-like tumor cells are further enriched in the hypoxic TME upon sequential implantation in vivo

**Fig. 4:**
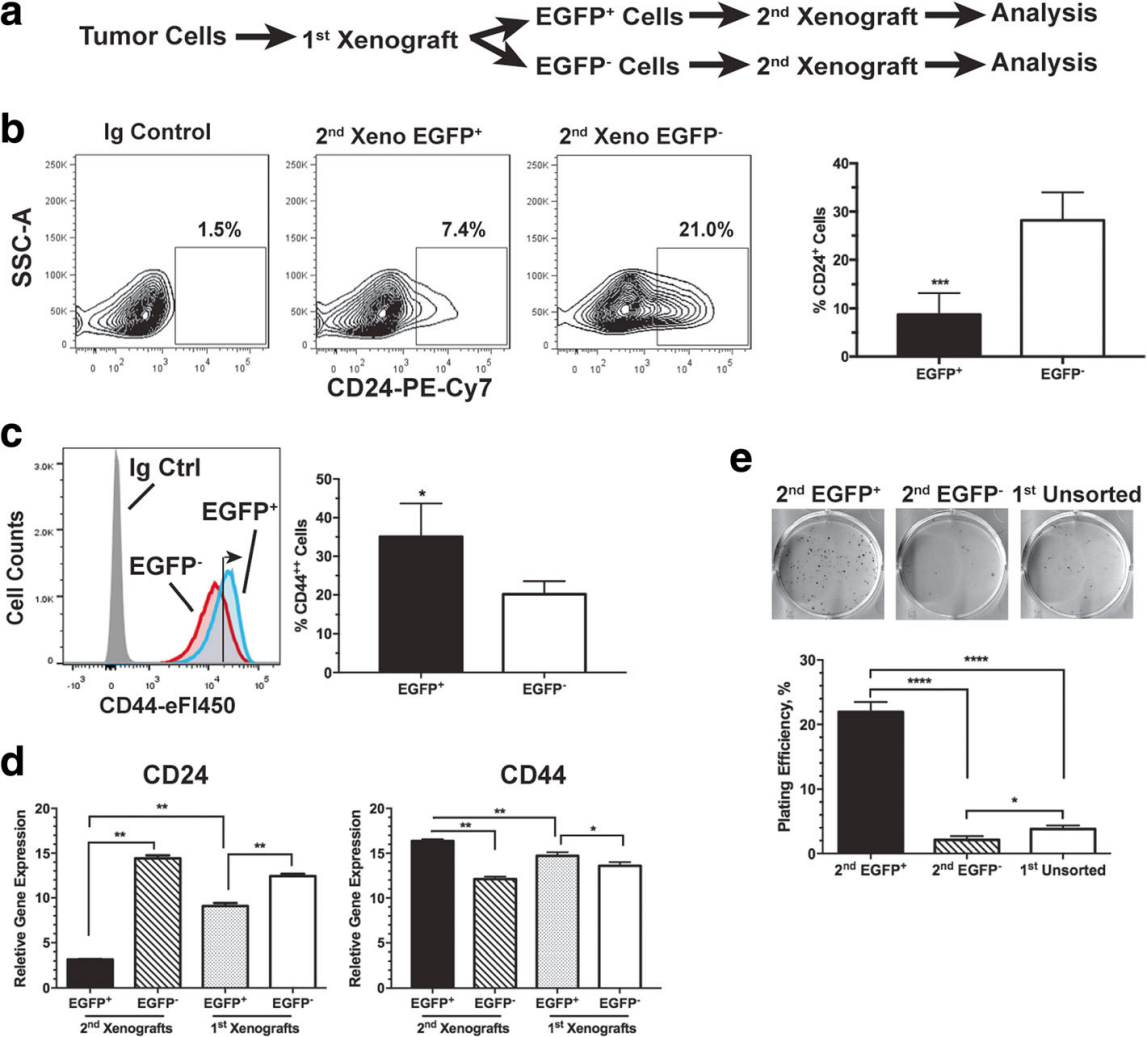
The cancer stem cell (CSC)-like population is further enriched in secondary xenografts derived from the enhanced green fluorescent protein (EGFP)^+^ MDA-MB-231 cells. **a** Generation of the secondary xenografts. **b**, **c** Surface levels of CD24 and CD44 are analyzed by fluorescence-activated cell sorting. **b** Average CD24^+^ populations from six individual tumors (***p < 0.001, Student’s *t* test). **c** Average CD44^++^ (right-pointing arrow) populations from three individual tumors (*p < 0.05, Student’s *t* test). **d** Quantitative RT-PCR analysis of expression of CD24 and CD44 genes in the EGFP^+^ and EGFP^−^ cells freshly isolated from either the 2^nd^ or 1^st^ xenografts (n = 3; *p < 0.05, **p < 0.01, Student’s *t* test). **e** Clonogenic growth of sorted EGFP^+^ and EGFP^−^ cells freshly isolated from the 2^nd^ xenografts in comparison to the unsorted tumor cells from the 1^st^ xenografts (n = 3; *p < 0.05, ***p < 0.0001, Student’s *t* test)

The results described above led to an interesting question as to whether the CSC characteristics continue to evolve upon repeated exposure to hypoxia in vivo, an important issue germane to the understanding of TME-driven malignant progression. Hence, we developed a model (Fig. 4a) in which we re-implanted freshly sorted EGFP^+^ hypoxic and EGFP^−^ non-hypoxic MDA-MB-231 tumor cells isolated from the 1^st^ xenograft tumor (1^st^ EGFP^+^ and 1^st^ EGFP^−^), respectively, in tumor-naive female nude mice. In order to maintain relative consistency of the tumor microenvironment, breast tumor cells isolated from orthotopic xenografts were re-implanted into the mammary fat pads while tumor cells isolated from subcutaneous sites were re-implanted ectopically.

As shown in Fig. 4b, the difference in the CD24^+^ population is further increased between the 2^nd^ xenografts derived from the 1^st^ sorted EGFP^+^ tumor cells and those derived from 1^st^ sorted EGFP^−^ cells. In particular, the CD24^+^ population increased from approximately 13% (Fig. 2b) to approximately 25% (Fig. 4b) when the EGFP^−^ MDA-MB-231 cells were re-implanted. Although both 2^nd^ xenografts maintained high levels of CD44, the xenografts derived from the EGFP^+^ cells had slightly but significantly higher levels of CD44 than the EGFP^−^ cell-derived xenografts (right arrow in the FACS histogram, Fig. 4c). The differential changes in cell surface expression of CD44 and CD24 are consistent with their differential gene expression between the two types of 2^nd^ xenografts and between the 2^nd^ and 1^st^ xenografts (Fig. 4d and Additional file 5). Functionally, the 2^nd^ xenograft tumor cells originating from the 1^st^ sorted EGFP^+^ cells displayed significantly higher clonogenic potential than the 2^nd^ xenograft tumor cells originating from the 1^st^ sorted EGFP^−^ cells and the unsorted tumors cells isolated from the 1^st^ xenografts (Fig. 4e). Collectively, these data suggest that the EGFP^−^ cells isolated from the non-hypoxic TME are likely to be more prone to differentiation whereas the EGFP^+^ hypoxic tumor cells continue to maintain their CSC-like cell fate and their aggressive properties.

**Fig. 5:**
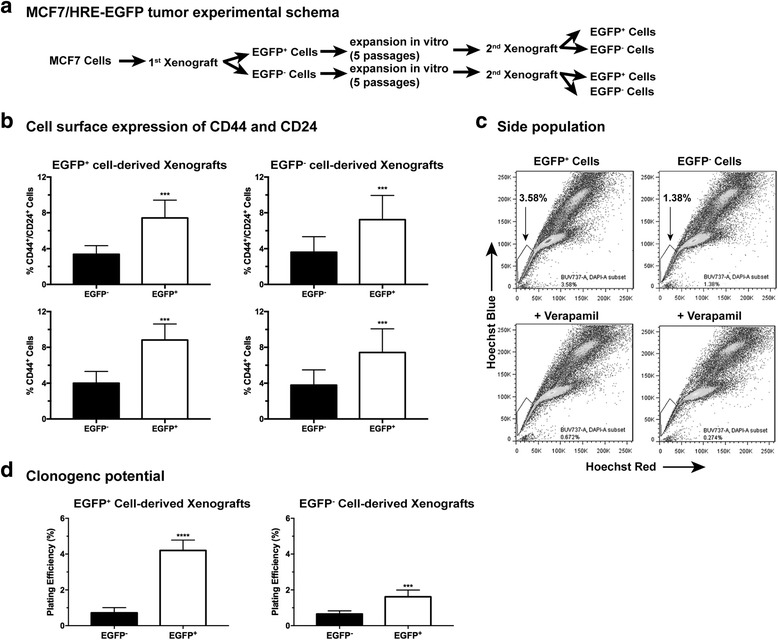
The cancer stem cell (CSC)-like characteristics of tumor cells isolated from the secondary enhanced green fluorescent protein (EGFP)^+^ MCF7/HRE-EGFP xenografts. **a** Generation of secondary MCF7/HRE-EGFP xenografts by re-implantation of sorted EGFP^+^ and EGFP^−^ cells isolated from the primary MCF7/HRE-EGFP xenografts. Tumor cells freshly isolated from the secondary xenografts were sorted into EGFP^+^ and EGFP^−^ populations for (**b**) fluorescence-activated cell sorting (FACS) analysis of the CD44^+^/CD24^+^ and CD44^+^ populations (*n* = 5 for EGFP^+^ cells, *n* = 4 for EGFP^−^ cells; ***p < 0.001, Student’s *t* test). **c** Side population (SP) of the secondary xenograft-derived tumor cells. MCF7 tumor cells were isolated from the secondary xenografts derived from EGFP^+^ and EGFP^-^ tumor cells, respectively, and expanded in vitro for three passages. Cells were stained with Hoechst 33342 for side population analysis by FACS. Verapamil (50 μM) was used to block nuclear export of Hoechst 33342. These results were validated in two independent experiments. **d** Clonogenic potential of the freshly sorted EGFP^+^ and EGFP^−^ populations from the secondary xenografts (*n* = 6; ****p < 0.0001, ***p < 0.001, Student’s *t* test)

As an independent model, we established 2^nd^ xenografts using EGFP^+^ and EGFP^−^ tumor cells *ex vivo* from the 1^st^ MCF7 orthotopic xenografts (Fig. 5). In contrast to the basal-like MDA-MB-231 cells, the luminal-like MCF7 cells do not experience significant changes in the CD44^+^ or CD44^+^/CD24^+^ phenotype upon re-implantation of the sorted hypoxic (EGFP^+^) or non-hypoxic (EGFP^−^) cells. Nonetheless, the CD44^+^ CSC-like cells continue to be significantly enriched in the hypoxic (EGFP^+^) population of tumor cells *ex vivo* from 2^nd^ xenografts derived from either EGFP^+^ or EGFP^−^ MCF7 cells (Fig. 5b). Consistently, the side population (SP) were also enriched in the 2^nd^ xenografts derived from EGFP^+^ MCF7 cells compared to those from EGFP^−^ cells (Fig. 5c). Functionally, EGFP^+^ MCF7 cells ex vivo from the 2^nd^ xenografts exhibit significantly higher clonogenic potentials than their EGFP^−^ counterparts from the same tumor (Fig. 5d). Together, these findings strongly suggest that selective pressures in the hypoxic TME favor the CSC-like cell fate of breast cancer cells independent of the tumor grade or type.

### The PI3K-AKT pathway is required for maintenance of the CD44^+^/CD24^−^ CSC phenotype

Upon examination of several stem-cell-related signaling pathways, we found that PI3K/AKT pathway, a critical pathway involved in pro-survival and pro-stem-cell maintenance [48–52], was preferentially activated in the ex vivo hypoxia-selected breast cancer cells. We found that the 2^nd^ xenograft tumor cells originating from the 1^st^ EGFP^+^ MDA-MB-231 cells showed robust AKT S473 phosphorylation in response to serum stimulation, compared to the 2^nd^ xenograft tumor cells originating from the 1^st^ EGFP^−^ cells (Additional file 6, panel A). Similar results were obtained from the 2^nd^ xenograft tumor cells derived from 1^st^ EGFP^+^ and EGFP^−^ MCF7 cells (Additional file 6, panel B). Because the preferential AKT activation in the ex vivo hypoxic cells was stably maintained even under the ambient non-hypoxic conditions, these data suggest that these hypoxic breast cancer cells isolated ex vivo from xenografts may have acquired a distinct and stable phenotype compared to their neighboring non-hypoxic tumor cells.

**Fig. 6:**
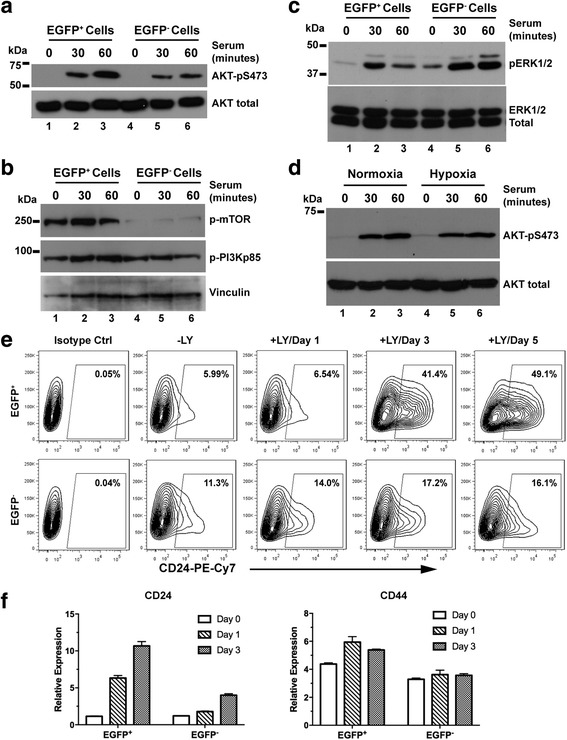
The PI3K/AKT pathway is required for maintenance of the CD44^+^/CD24^−^ cancer stem cell (CSC) phenotype. The enhanced green fluorescent protein (EGFP)^+^ and EGFP^−^ cells sorted from the 1^st^ MDA-MB-231 xenografts underwent serum starvation overnight. After serum stimulation, phosphorylation of AKT (**a**), PI3Kp85 (**b**), mTOR (**b**), and ERK1/2 (**c**) was analyzed using Western blots. **d** Serum-stimulated AKT phosphorylation in the parental MDA-MB-231/HRE-EGFP cells under normoxia and hypoxia (1% O_2_). **e** Increase in the CD24^+^ population induced by the PI3K inhibitor LY294002 (20 μM). **f** Quantitative RT-PCR analysis of expression of CD24 and CD44 genes in response to LY294002. These observations are confirmed by independent experiments

To further understand the stable phenotypes of the hypoxic TME-selected tumor cells, we established short-term in vitro cultures of the 1^st^ xenograft-derived EGFP^+^ and EGFP^−^ cells and interrogated the PI3K-AKT pathway that is strongly implicated in stem cell regulation and malignant progression including that of breast cancers [48–52]. Following serum starvation and then stimulation in vitro, phosphorylation of AKT is much more strongly induced in the EGFP^+^ MDA-MB-231 cells than in the EGFP^−^ cells (Fig. 6a). The differences in mTOR phosphorylation are also apparent with constitutively high levels of mTOR phosphorylation in the EGFP^+^ MDA-MB-231 cells but much reduced mTOR phosphorylation in the EGFP^−^ cells (Fig. 6b). Relatively smaller changes are found in phosphorylation of the PI3Kp85 subunit (Fig. 6b). In contrast, the serum-induced phosphorylation of ERK1/2 is somewhat reduced in the EGFP^+^ MDA-MB-231 cells (Fig. 6c). Importantly, enhanced AKT phosphorylation was observed only in the *ex vivo* EGFP^+^ MDA-MB-231 cells derived from xenografts; exposure of MDA-MB-231 cells to hypoxia in vitro, by itself, did not significantly affect AKT phosphorylation in response to serum stimulation (Fig. 6d). Under the same in vitro conditions, the cellular microenvironment remained the same for both the xenograft-derived EGFP^+^ and EGFP^−^ cells. Furthermore, the HIF-1α and HIF-2α proteins become destabilized ex vivo in both EGFP^+^ and EGFP^−^ tumor cells under the ambient tissue culture conditions (Additional file 2, pannel A), suggesting that the differential activation of the PI3K-AKT pathway that persists in cell culture after isolation from the xenografts is independent of HIF-1/2. The differential activation of the PI3K/AKT pathway exhibited by the ex vivo EGFP^+^ and EGFP^−^ tumor cells thus clearly illustrates the phenotypic differences between these two ex-vivo derived populations of breast cancer cells localized in two different compartments of the TME, which is consistent with their corresponding CSC-like characteristic shown in Figs. 2, 3, 4 and 5. These stable phenotypic differences suggest that breast cancer cells undergo clonal evolution and/or selection in the hypoxic TME that leads to acquisition of new and sustained clonal properties.

The PI3K-AKT pathway is well-recognized as a key mechanism for regulating maintenance, proliferation and survival of both normal and cancer stem cells [53–55]. We examined whether the PI3K/AKT pathway plays an important role in the maintenance of the CSC-like phenotype of the ex vivo EGFP^+^ hypoxic tumor cells using LY294002, a specific PI3K inhibitor, to suppress AKT activation (Additional file 2, pannel B). After incubation with LY294002 for up to 5 days in vitro, both the xenograft-derived EGFP^+^ and EGFP^−^ MDA-MB-231 cells showed progressive increases in the CD24^+^ population (Fig. 6e), indicating cell differentiation. Notably, the EGFP^+^ cells showed stronger upregulation of CD24. In contrast, CD44 expression was not significantly affected in either cell population (data not shown). The increase in the CD24^+^ population correlated with increased CD24 gene expression, whereas there were relatively small changes in CD44 gene expression (Fig. 6f). Although the EGFP^+^ tumor cells displayed robust mTOR phosphorylation, the mTOR inhibitor, rapamycin, did not strongly affect the CSC-associated CD24 and CD44 markers (data not shown). Collectively, these data suggest that the enhanced AKT activation is required for maintaining the CSC-like phenotype exhibited by the EGFP^+^ breast cancer cells isolated ex vivo from xenografts.

## Discussion

The hypoxia-dependent regulation of CSC-associated phenotypes and functions has been proposed and actively investigated [7, 39–41]. A number of in vitro studies have shown that hypoxia or hypoxia-sensing pathways play a significant role in the maintenance of the CSC phenotype in breast cancer cells [56–62]. Hypoxia is also implicated in increased CSC-like populations in breast cancer xenografts treated by antiangiogenic agents [63]. However, definitive in vivo evidence has remained elusive. Herein, our data have provided direct evidence demonstrating that the hypoxic TME favors enrichment and/or maintenance of the CSC-like tumor cells in vivo.

In this study, we have established hypoxia-sensing xenograft models using the 5xHRE-driven d2EGFP reporter gene (HRE-EGFP) [27] in breast cancer cell lines. In the xenografts derived from these breast tumor cell lines, the EGFP^+^ tumor cells are primarily localized in the regions that are positively stained for pimonidazole, a widely used bioreductive marker of hypoxia. These results indicate that our HRE-EGFP-based xenograft models can reliably identify tumor cells that are hypoxic in the TME. Because the expression of d2EGFP (half-life ≅ 2 h) requires stabilized HIF-1α and/or HIF-2α, these models are best suited for identifying hypoxic tumor cells around the time of observation or analysis. Similar strategies have been used in other tumor models [30, 31] to identify hypoxic cells. Nonetheless, we cannot rule out the possibility that some tumor cells might express EGFP due to a condition of pseudohypoxia, i.e. stabilization of HIF-α without oxygen deficiency.

As shown by our combined in vitro and in vivo data, our xenograft model has the potential to identify hypoxic tumor cells at approximately ≤ 10 mmHg pO_2_ or ≤ 1.3% O_2_, which is well within the hypoxic range in human breast cancers clinically observed by direct pO_2_ measurement [64]. However, identification and isolation of viable hypoxic breast cancer tumor cells in the clinics are extremely challenging, if possible at all. Nonetheless, a comprehensive approach using both genomics and proteomics could be employed to determine whether breast cancer stem cells are enriched in the hypoxic regions of clinical human breast cancers.

Different breast cancer cell lines express different sets of cancer stem cell markers, which may reflect their respective status of development or differentiation. Nonetheless, a number of studies have shown that CD44 expression is preferentially associated with the undifferentiated or progenitor-like phenotype whereas CD24 is expressed in more differentiated breast cancer cells with a luminal phenotype [65, 66]. MDA-MB-231 cells maintain high levels of CD44. Increased CD24 expression could signal differentiation. Our data indeed confirm that the CD44^+^/CD24^−^ MDA-MB-231 cells exhibit stronger stem cell characteristics than their CD44^+^/CD24^+^ counterparts do. Therefore, we consider the CD44^+^/CD24^−^ MDA-MB-231 cells as being more breast cancer stemcell-like. On the other hand, MCF7 cells are mainly CD44^−/low^/CD24^+^. Emergence of CD44^+^/CD24^+^ MCF7 cells, also positive for another stem cell marker CD49f, could result from increased stemcell-like populations in the hypoxic tumor microenvironment in vivo. Importantly, the ex vivo hypoxic tumor cells from both breast cancer xenograft models possess biological functions closely associated with CSC characteristics, including self-renewal and clonogenic potential, motility and invasion, and tumorigenicity. It is noteworthy that the enrichment of the CSC-like population occurs in the hypoxic TME in vivo only and exposure to hypoxia in vitro does not significantly affect cell fate. Consistent with this point of view, a comprehensive bioinformatics study [67] has shown that the hypoxia gene signatures obtained from various in vitro cell culture models have poor prognostic values when applied to large clinical datasets of breast cancer patients. In contrast, the patient tumor-derived hypoxia gene signatures have statistically significant prognostic values [67]. These findings strongly suggest that the phenotype and cell fate of tumor cells located in the naturally hypoxic regions are likely to be determined collectively by multiple factors in the hypoxic TME in combination with O_2_ deficiency.

Interestingly, the CSC characteristics of the EGFP^+^ hypoxic cells, but not the EGFP^−^ non-hypoxic cells, are further increased upon re-implantation. These observations suggest that tumor cells localized in the hypoxic TME might have acquired relatively stable phenotypes especially those closely associated with the CSC characteristics and that they continue to evolve upon re-implantation. Consistent with this new concept, we have found that the ex vivo EGFP^+^ hypoxic cells exhibit functionally distinct cell signaling pathways, including the PI3K/AKT pathway, from the EGFP^−^ non-hypoxic cells even when they are maintained under the same ambient culture conditions. Although AKT activation in breast cancer cell lines has also been reported under in vitro hypoxic conditions [63], we have found in this study that hypoxia in vitro alone is not sufficient to alter these signaling pathways, suggesting the new cellular phenotype exhibited by the ex vivo EGFP^+^ hypoxic cells most likely results from complex regulations in the hypoxic TME. Nonetheless, these data strongly suggest that the hypoxic TME has the potential to cause tumor cells to evolve and to acquire new and stable properties that are distinct from those of tumor cells localized in the non-hypoxic TME within the same tumor mass. Consistent with these findings, it has recently been shown that the hypoxic TME can give rise to a subpopulation of tumor cells with a dormancy phenotype that is maintained even after dissemination [30].

As we have found, the ex vivo EGFP^+^ hypoxic cells show robust AKT activation in response to serum stimulation even under non-hypoxic conditions compared to the ex vivo EGFP^−^ non-hypoxic cells that exhibit weak to moderate response. This unique phenotype has potentially significant biological implications because a similar situation of serum starvation-stimulation could be encountered in vivo. Hypoxic areas in solid tumors are often poorly perfused due to compromised blood flow and vascular malfunctions. Therefore, concentrations of serum-derived growth factors and other nutrients are expected to be much lower in the hypoxic areas than those in the well-perfused non-hypoxic regions. Serum stimulation occurs when a previously hypoxic area becomes re-oxygenated or when a hypoxic tumor cell invades locally into a non-hypoxic area or enters the bloodstream. The preferential activation of the PI3K/AKT pathway may confer significant survival and/or growth advantages on the previously hypoxic tumor cells during their invasion and metastasis.

We have further found that pharmacological inhibition of the PI3K/AKT pathway leads to strong increases in the CD24^+^ population of MDA-MB-231 cells, suggesting that activity of the PI3K/AKT pathway is essential for the maintenance of their CSC phenotype. In comparison to the ex vivo EGFP^−^ cells, the CSC phenotype of the EGFP^+^ hypoxic cells is much more sensitive to inhibition of PI3K/AKT. This observation is consistent with the well-recognized role of the PI3K/AKT pathway in the maintenance of both normal and cancer stem cells [53–55]. Nonetheless, it is highly possible that other as yet unidentified stem-cell-related pathways may also be involved in cell fate determination by the hypoxic tumor microenvironment.

## Conclusion

Cancer cell stemness, especially the capacity of self-renewal, is essential for enabling malignant progression and clonal evolution. In this study, we have provided direct evidence demonstrating that the hypoxic TME in vivo favors the enrichment and/or selection of the CSC-like characteristics. Importantly, the differential phenotypes of the tumor cells ex vivo from the hypoxic and non-hypoxic TME, respectively, are relatively stable even when they are subsequently maintained under normoxic ambient culture conditions, which suggests active clonal evolution and/or selection to yield a durable phenotype in the hypoxic TME in vivo.

In light of the findings that hypoxia occurs in human breast cancer [64] and is associated with poor treatment outcomes [68], the novel observations presented in this study strongly suggest that the hypoxic TME promotes malignant progression and confers resistance to therapy, at least in part, by inducing or sustaining the cancer stem cell phenotype.

## Additional files

Additional file 1: **Figure S1.** The hypoxia-sensing human breast cancer xenograft model. A FACS analysis of EGFP^+^ populations in the selected MDA-MB-231 cells stably expressing the HRE-EGPF reporter gene after exposure to hypoxia in vitro at 1% O_2_. B Co-localization of the EGFP^+^ tumor cells with immunofluorescent stains (red) of the Hypoxyprobe in MCF7/HRE-EGFP xenografts. (TIFF 9624 kb)

Additional file 2: **Figure S2.** Examination of HIF-1α and HIF-2α in sorted tumor cells from the MDA-MB-231/HRE-EGFP xenografts, and inhibition of AKT phosphorylation by LY294002. (A) Dynamic regulation of HIF-1α and HIF-2α proteins in response to hypoxia and re-oxygenation is examined in Western blots. These results show that the O_2_-dependent regulation of HIF-α stability in the sorted EGFP^+^ and EGFP^−^ tumor cells is normal and comparable to the parental MDA-MB-231 cells. (B) The PI3K-specific inhibitor LY294002 (20 μM) blocks AKT phosphorylation in the sorted EGFP^+^ and EGFP^−^ MDA-MB-231 cells. (TIFF 2045 kb)

Additional file 3: **Figure S3.** Characterization of the sorted EGFP^+^ and EGFP^−^ cells freshly isolated from the MDA-MB-231/HRE-EGFP xenografts. (A) Purification of MDA-MB-231 cells from xenografts. The xenografts contain approximately 75% human tumor cells, based on cell surface expression of CD326 (human EpCAM). After depletion of mouse cells, purity of tumor cells reaches 98%. (B) Expression of CSC-related markers, CD24 and CD44, and hypoxia-induced genes, LOX1 and GLUT1, is analyzed by qRT-PCR. EGFP^+^ and EGFP^−^ cells are freshly isolated from both orthotopic and ectopic xenografts, respectively (*n* = 3–5; *p < 0.05, **p < 0.01, Student’s *t* test). Gene expression is not affected by tumor sites. (C) Side population (SP) of freshly isolated MDA-MB-231 cells from orthotopic xenografts. The unsorted tumor cells were stained with Hoechst 33342. The entire tumor cell populations were then gated into the EGFP^+^ and EGFP^−^ subpopulations, respectively, for side population analysis by FACS. Verapamil (50 μM) was used to block nuclear export of Hoechst 33342. These results were validated in three independent experiments. (TIFF 13956 kb)

Additional file 4: **Figure S4.** Tumor sphere formation and clonogenic growth of sorted EGFP^+^ and EGFP^−^ cells freshly isolated from mouse 4T1/HRE-EGFP allogafts. The 4T1/HRE-EGFP cell line is established using the same approach as that for MDA-MB-231 and MCF7 cell lines. Allografts are generated by injection of 4T1/HRE-EGFP tumor cells either in the mammary fat pads (orthotopic) or in the hind back (subcutaneous) of female athymic mice. The EGFP^+^ and EGFP^−^ tumor cells are sorted by FACS from enzymatically dissociated tumor mass. (A) The self-renewal potential is evaluated using the tumor sphere formation assay (*n* = 6; **p < 0.01, ***p < 0.001, Student’s *t* test). (B) Clonogenicity is examined by plating the sorted cells at a clonal density (300 cells/well in 6-well plates, n = 6; ****p < 0.0001, Student’s *t* test). (TIFF 1025 kb)

Additional file 5: **Figure S5.** The CSC-like characteristics of tumor cells isolated from the secondary MDA-MB-231/HRE-EGFP xenografts. (A, B) The secondary MDA-MB-231 xenografts are generated by re-implanting the sorted EGFP^+^ and EGFP^−^ tumor cells, respectively. Gene expression is analyzed by qRT-PCR (n = 3; *p < 0.05, **p < 0.01, ***p < 0.001, ****p < 0.0001, Student’s *t* test). (TIFF 1391 kb)

Additional file 6: **Figure S6.** Differential activation of AKT in sorted EGFP^+^ and EGFP^−^ cells isolated from xenografts. The EGFP^+^ and EGFP^−^ cells isolated ex vivo from xenografts are maintained in vitro for ≤ 5 passages. After overnight serum starvation, the tumor cells are stimulated with serum (10% FBS in culture medium). AKT phosphorylation is examined by Western blotting of whole cell extracts of tumor cells from the 2nd MDA-MB-231 (A) and MCF7/HRE-EGFP (B) xenografts, respectively. (TIFF 1903 kb)
