# Supplementary figures and images for: The hypoxic tumor microenvironment in vivo selects the cancer stem cell fate of breast cancer cells
Source: Breast Cancer Res. 2018 Mar 6;20:16. doi: 10.1186/s13058-018-0944-8 (PMC5840770; doi:10.1186/s13058-018-0944-8)

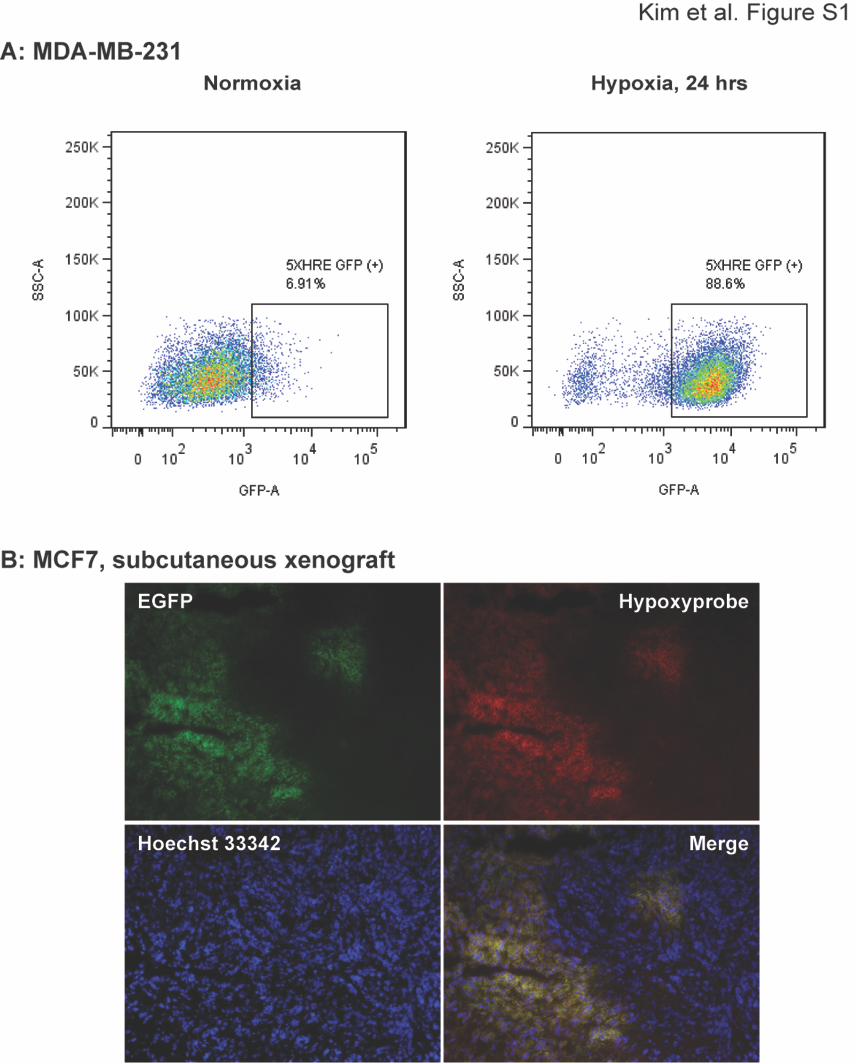

Supplement: Supplementary file 1 — Figure S1. The hypoxia-sensing human breast cancer xenograft model. A FACS analysis of EGFP+ populations in the selected MDA-MB-231 cells stably expressing the HRE-EGPF reporter gene after exposure to hypoxia in vitro at 1% O2. B Co-localization of the EGFP+ tumor cells with immunofluorescent stains (red) of the Hypoxyprobe in MCF7/HRE-EGFP xenografts. (TIFF 9624 kb) [file 13058_2018_944_MOESM1_ESM.tif]

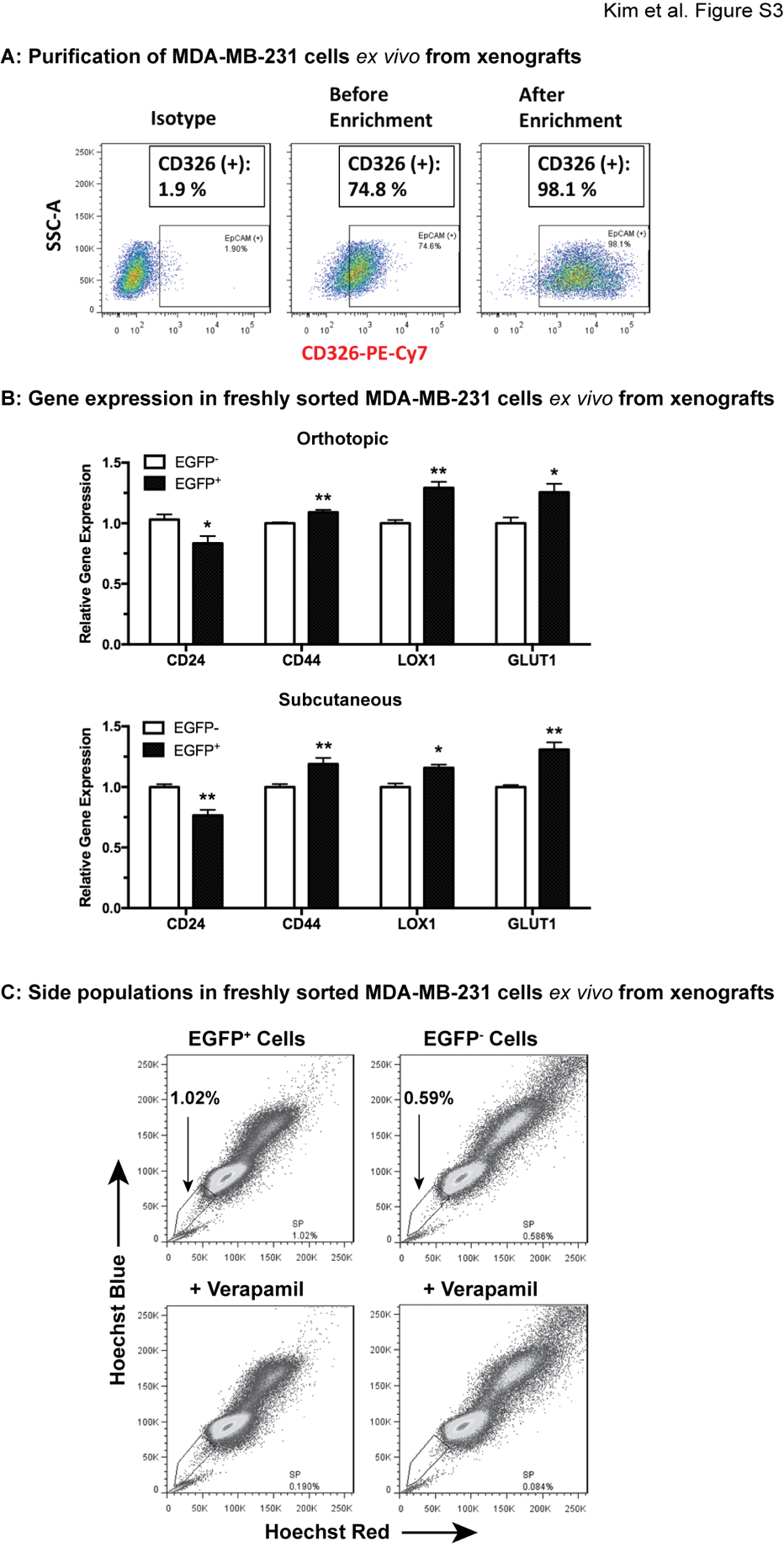

Supplement: Supplementary file 3 — Figure S3. Characterization of the sorted EGFP+ and EGFP− cells freshly isolated from the MDA-MB-231/HRE-EGFP xenografts. (A) Purification of MDA-MB-231 cells from xenografts. The xenografts contain approximately 75% human tumor cells, based on cell surface expression of CD326 (human EpCAM). After depletion of mouse cells, purity of tumor cells reaches 98%. (B) Expression of CSC-related markers, CD24 and CD44, and hypoxia-induced genes, LOX1 and GLUT1, is analyzed by qRT-PCR. EGFP+ and EGFP− cells are freshly isolated from both orthotopic and ectopic xenografts, respectively (n = 3–5; *p < 0.05, **p < 0.01, Student’s t test). Gene expression is not affected by tumor sites. (C) Side population (SP) of freshly isolated MDA-MB-231 cells from orthotopic xenografts. The unsorted tumor cells were stained with Hoechst 33342. The entire tumor cell populations were then gated into the EGFP+ and EGFP− subpopulations, respectively, for side population analysis by FACS. Verapamil (50 μM) was used to block nuclear export of Hoechst 33342. These results were validated in three independent experiments. (TIFF 13956 kb) [file 13058_2018_944_MOESM3_ESM.tif]
